# Responsible AI for Sepsis Prediction: Bridging the Gap Between Machine Learning Performance and Clinical Trust

**DOI:** 10.3390/jcm15062251

**Published:** 2026-03-16

**Authors:** Thiago Q. Oliveira, Leandro A. Carvalho, Flávio R. C. Sousa, João B. F. Filho, Khalil F. Oliveira, Daniel A. B. Tavares

**Affiliations:** 1 Department of Computer Science, Federal Institute of Ceará, Fortaleza 60040-531, CE, Brazil; 2 Department of Computer Science, Federal University of Ceará, Fortaleza 60440-900, CE, Brazil; 3 Emergency Department, General Hospital of Fortaleza, Fortaleza 60150-160, CE, Brazil

**Keywords:** sepsis, machine learning, artificial intelligence, responsible artificial intelligence, MIMIC-IV, intensive care unit

## Abstract

**Background:** Sepsis remains a leading cause of mortality in intensive care units (ICUs) worldwide. Machine learning models for clinical prediction must be accurate, fair, transparent, and reliable to ensure that physicians feel confident in their decision-making processes. **Methods:** We used the MIMIC-IV (version 3.1) database to evaluate several machine learning architectures, including Logistic Regression, XGBoost, LightGBM, LSTM (Long Short-Term Memory) networks and Transformer models. We predicted three main clinical targets—hospital mortality, length of stay, and septic shock onset—using artificial intelligence algorithms, with respect for responsible AI principles. Model interpretability was assessed using Shapley Additive Explanations (SHAP). **Results:** The XGBoost model demonstrated superior performance in prediction tasks, particularly for hospital mortality (AUROC 0.874), outperforming traditional LSTM networks, Transformers, and linear baselines. The importance analysis of the variables confirmed the clinical relevance of the model. **Conclusions:** While XGBoost and ensemble algorithms demonstrate superior predictive power for sepsis prognosis, their clinical adoption necessitates robust explainability mechanisms to gain trust among doctors.

## 1. Introduction

According to the World Health Organization [1], sepsis is one of the leading causes of death worldwide, with 48.9 million cases and 11 million deaths in 2020 alone, representing 20% of all deaths worldwide. In addition to the high mortality rate, sepsis treatment is excessively expensive, given the occupancy of intensive care unit (ICU) beds, long hospital stays, need for constant monitoring, and complex treatments. The average hospital-wide cost of sepsis has been estimated to be more than USD 32,000 per patient in high-income countries [2].

Sepsis-3, since the 2016 consensus conference, has been defined as an infectious episode leading to life-threatening organ dysfunction caused by inadequate host response to infection [3]. Although physicians have an abundance of information about patients in the ICU for the treatment of sepsis, such as laboratory results, imaging studies, and clinical documentation, this cognitive overload can often hinder the identification of relevant patterns and the decision-making process [4].

Artificial intelligence (AI) and machine learning (ML) have been demonstrated to have great potential for predicting, diagnosing, and providing individualized treatment for sepsis. In diagnosis, AI has been shown to have advantages over traditional sepsis classification systems, such as the Systemic Inflammatory Response Syndrome (SIRS), Sequential Organ Failure Score (SOFA), and quick SOFA (qSOFA) systems, as AI can identify sepsis several hours in advance [5,6]. Early detection allows clinicians to intervene faster, increasing the chances of successful treatment [7].

However, the “black-box” nature of advanced machine learning models (MLMs) poses a significant barrier to clinical adoption [8]. Machine learning models for clinical prediction must be accurate, fair, transparent, and reliable [9] so that physicians feel confident in their decision-making process [10]. Responsible AI practices are crucial for predicting sepsis, a disease with high mortality rates and length of hospital stay [11,12,13,14,15].

This study explored the prediction of mortality, length of stay, and septic shock in patients with sepsis using artificial intelligence algorithms, respecting the following responsible AI principles:

- Transparent and explicable: can physicians understand why a prediction was made?
- Trust: are the predictions reliable and free of data leaks?
- Fairness: do the models perform equally well in all patient subgroups?
- Privacy: are patient data handled responsibly?

In this study, we used a Sepsis-3 cohort extracted from MIMIC-IV [16], version 3.1 [17]. The dataset contains 35,215 ICU admissions that met the criteria for Sepsis-3, with selected and time-aligned characteristics, including vital signs, laboratory test results, and therapeutic interventions, such as vasopressors, intravenous fluids, and antibiotics.

## 2. Materials and Methods

In this study, we used machine learning (ML) for the prediction of mortality, length of stay, and septic shock in patients with sepsis, respecting the principles of responsible AI. Our approach was developed following the Transparent Reporting of a Multivariable Prediction Model for Individual Prognosis or Diagnosis—Artificial Intelligence (TRIPOD-AI) reporting guidelines [18], aligning with the standards that balance innovation with clinical responsibility, as suggested in recent editorials [19].

This study was developed based on a public benchmark on Sepsis-3 using the MIMIC-IV database, version 3.1, which is a large, freely available database comprising de-identified health-related data associated with patients who stayed in the critical care units of the Beth Israel Deaconess Medical Center [20]. Paperpal, an artificial-intelligence-based tool, was used to support the writing and the review of English grammar in this study.

### 2.1. Data Source

The MIMIC-IV database, version 3.1, officially released in October 2024, was used. It contains hospitalization data for 364,627 unique patients between 2008 and 2022. This database aligns with ethical and safety principles, as its structure and governance incorporate many ethical pillars required for the development of safe healthcare systems.

Privacy and security principles are guaranteed when providing an anonymized database, meaning that the personal identifiers of patients and healthcare professionals have been removed or altered to protect their identity, in accordance with the Health Insurance Portability and Accountability Act (HIPAA). Unlike open databases, access to MIMIC-IV requires researchers to complete research ethics training provided by the PhysioNet platform and sign a Data Use Agreement (DUA). The PhysioNet platform is managed by members of the MIT Laboratory for Computational Physiology [17,21].

MIMIC-IV promotes the principle of transparency by making the code for the data processing scripts (SQL and Python) available on the GitHub platform, allowing the scientific community to audit and reproduce the results. Furthermore, the community is encouraged to contribute to the project, either by proposing improvements or reporting possible errors.

### 2.2. Cohort Selection

This section describes how the cohort was generated by the authors. The cohort process was based on methodologies from prior sepsis studies [22] that preserved the temporal structure of the clinical trajectories. The data were extracted and grouped into categories: (1) demographics, such as age, sex, and gender; (2) clinical measurements, such as laboratory tests and vital signs; and (3) clinical interventions, such as vasopressors, fluids, and mechanical ventilation.

To identify suspected infection, the Sepsis-3 definition was applied [3], using either antibiotic administration records or positive microbiological cultures as potential infection triggers, i.e., the earliest time point where a patient’s SOFA Score increased by two or more points from baseline. A time window of 24 h before and 72 h after the onset of sepsis was defined. This window aims to capture early-detection signs up to the acute phase of infection. The clinical measurements in this window were standardized, and outliers were removed. The data were regrouped at 4-h intervals. The mean was computed when multiple values were present within an interval.

The missing data shortage was addressed using a forward-filling imputation strategy to prevent temporal data leakage and ensure real-world clinical deployability [23]. For any missing value at a given time step, the model only propagates the most recent valid measurement recorded for that specific patient. If a variable lacks historical data at the absolute beginning of a patient’s trajectory, it is initialized using the global baseline median computed solely from the cross-validation training fold, ensuring no future information is ever exposed.

Variables with more than 80% of data missing were removed to avoid the risk of introducing bias when performing imputation. Some variables are estimated based on clinical rules, such as FiO_2_, which is derived from the oxygen flow rate and device type, and the Glasgow Coma Scale (GCS), which is calculated from the RASS Score [24]. The SOFA, NEWS, and SIRS Scores were calculated from the cleaned data.

Four types of central interventions for the treatment of sepsis were extracted: (1) mechanical ventilation (mode and parameters), (2) antibiotics (timing and number of unique agents), (3) fluid resuscitation (standardized to 0.9% NaCl equivalent volume), and (4) vasopressors (converted to norepinephrine-equivalent dosage). Every 4 h, the cumulative vasopressor levels, doses, and volumes were recorded. These variables allow us to observe not only the patient’s status but also the dynamics of the treatment.

Septic shock is defined using three conditions: (1) administration of at least 2000 mL of fluids in the prior 12 h, (2) MAP < 65 mmHg despite fluid resuscitation, and (3) vasopressor requirement with lactate > 2 mmol/L [3,25]. Patients under 18 years of age, patients with insufficient data, and individuals who died shortly after admission to the ICU were excluded, as there was not enough time for adequate treatment to be administered.

### 2.3. Data Preprocessing

Clinical data were extracted and processed into time series formats using the reproducible pipeline established by [20]. After review by clinicians, the NEWS Score was calculated and added [26] to this pipeline; this is an important score in identifying sepsis early [27].

Table 1 lists the predictor variables used in the tasks, excluding identifiers and target variables such as mortality, length of stay (LOS), and septic shock. The predictor variables were grouped into the following categories: demographic data, vital signs, laboratory tests, hematological tests, arterial blood gas analysis, ventilation and oxygenation, liver function, clinical scores, and fluid administration.

In total, the cohort contained 78 predictor variables, some of which were derived from other information, such as score variables like NEWS, and some variables could be considered redundant, such as temp_C (temperature Celsius) and temp_F (temperature Fahrenheit). The complete list in alphabetical order is presented in Table A1.

Records with outliers were excluded. The initial cohort contained 36,613 records identified as sepsis. Within this set, 13 records were excluded due to extreme urine values, exceeding 12.000 mL within a 4-h period, possibly due to typing or sensor errors. Twelve records of patients who received over 10.000 mL of fluids within a 4-h period were also excluded.

A total of 870 records were excluded due to premature death occurring within less than 24 h after ICU admission, as these patients were considered to have advanced sepsis, and the model would not be able to systematically track the progression of the condition, potentially introducing future bias. A further 503 records were excluded because they did not have a SOFA Score greater than 2 throughout their hospital stay, failing to meet the Sepsis-3 criteria. Therefore, 1398 records were excluded, resulting in a final model with 35,215 records.

To prevent post-treatment bias (or label leakage), a phenomenon where predictive algorithms learn to forecast clinical deterioration based on therapeutic interventions, all treatment-guided variables were excluded from the final modeling feature space. Predictors pertaining to hemodynamic support (vasopressor presence and dose), respiratory interventions (mechanical ventilation, PEEP, tidal and minute volumes, and airway pressure), fluid resuscitation volumes, and antibiotic administration logs were excluded.

### 2.4. Data Statistics

Table 2 presents the cohort characteristics. The average age was 65 years, with a standard deviation of 16.3 years, and 38.1% of the patients were between 41 and 65 years old. There was a relative predominance of male patients (approximately 55%). The median Charlson Comorbidity Index of 5.0 suggests a considerable burden of comorbidities in this cohort. According to the BMI criteria, more than 60% of the patients were overweight or obese.

The mortality rate during hospitalization was 14.5%. However, mortality within 90 days was 25.5%, reflecting the difference between patients who were discharged alive but subsequently died. The average length of ICU stay (LOS) was 5.1 days, with a standard deviation of 7.1 days. The standard deviation (7.1) was greater than the mean (5.1), confirming that the distribution was very asymmetrical (some patients stayed in hospital for a long time, pulling the mean upwards). The median (2.65 [1.37–5.73]) was the best measure of central tendency for the LOS.

The mean SOFA Score of 5.5 (±2.8) reflects substantial organ dysfunction, typical of patients who meet the Sepsis-3 criteria, indicating a significant but not extreme risk of in-hospital mortality. The mean NEWS Score of 6.06 (±2.57) indicates a medium-to-intermediate risk of clinical deterioration in a patient. This score indicates that the patient’s vital signs vary significantly from normal levels and require frequent monitoring.

The mean values of the Glasgow Coma Scale (GCS) and RASS scores were consistent with septic conditions, indicating that these patients frequently have altered mental status or are sedated, and may also be drowsy. Most patients were treated with antibiotics (66.3%), while 35.1% required mechanical ventilation and 16.9% used vasopressors.

Table 3 presents the baseline characteristics of the survivors and non-survivors. Most variables showed statistically significant differences (p<0.001). Non-survivors were older and had a higher burden of comorbidities, as shown by the Charlson Index. Admission severity scores, including SOFA and NEWS Scores, were significantly elevated in this group. Laboratory markers also confirmed the presence of greater organ dysfunction. In contrast, sex and temperature did not differ significantly between the outcomes.

According to Table 3, the difference in median SpO_2_ (97.2% for survivors vs. 96.9% for non-survivors) is statistically significant, but has no practical clinical relevance, since both values represent normal physiological oxygenation. Owing to the large sample size (*n* = 35,215), small variations between groups can reach high statistical significance (p<0.001). Therefore, it is important to distinguish between clinical and statistical significance. On the other hand, the variations in values between the two groups in SOFA Score, age, and lactate levels represent high statistical and clinical significance, accurately reflecting the severity of the disease.

To statistically validate the results, a 95% Confidence Interval (CI) was used, calculated via bootstrap over 1000 iterations. The statistical significance between the AUROC of all models was assessed using the DeLong test. The Brier Score and Expected Calibration Error (ECE) were also used.

### 2.5. Machine Learning Models

**Figure 1:**
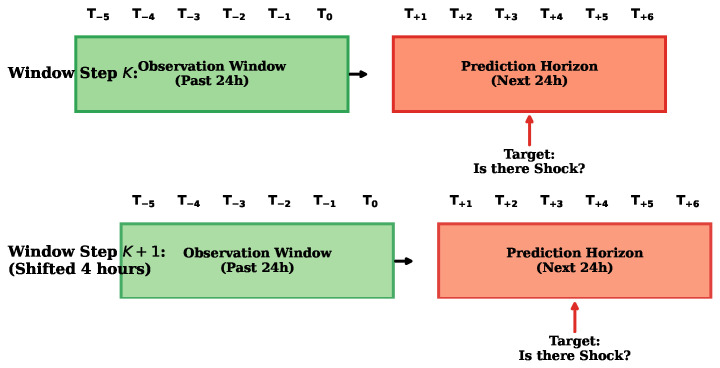
Dynamic sliding window for septic shock prediction. At each window step *K*, observation window features (past 24 h) were extracted to predict whether septic shock would occur at any point during the prediction horizon (next 24 h). The timeline illustrates the 4-h temporal shift between steps *K* and K+1.

This study evaluated predictive models across three critical clinical tasks in the context of sepsis: mortality prediction, length of stay (LOS), and septic shock. These tasks were grouped into two categories based on their temporal structures:

1. Static prediction: For mortality and LOS, the models uses only the first 24 h after sepsis detection. Clinical data were aggregated into 4-h time steps, and six time steps (equivalent to 24 h) of data were used to make predictions. We represent the time steps for T−N, where N represents the time steps; for example, T−0 represents the more recent moment and T−5 represents five time steps before (between 20 h and 24 h).
2. Dynamic prediction with a sliding window: For septic shock, the model takes the last 24 h (window_size = 6) and tries to predict whether an event (septic shock) will occur in the next 24 h (prediction_horizon = 6), as illustrated in Figure 1. Therefore, the model learns to predict a septic shock event at any point during hospital stay by looking at the recent 24-h history. Given our 78 discrete clinical features over a sliding 6-h observation window, each temporal prediction step consists of a simplified feature space of exactly 468 input variables (78 × 6 = 468).
  To avoid patient-level data leakage, all data splitting is performed at the patient-stay level using a Stratified Group K-Fold cross-validation strategy. This ensures that all sliding windows generated from a single patient’s trajectory are assigned entirely to either the training or the validation fold.

Table 4 summarizes the prediction tasks, modeling setup, and evaluation metrics. Mortality and septic shock prediction are defined as binary classification problems and LOS as a regression. We standardized the metrics across tasks to enable consistent and fair comparisons between the models.

For the mortality and septic shock tasks, we used binary classification to predict the probability of event occurrence. To evaluate these models, we employed the Area Under the Receiver Operating Characteristic curve (AUROC) and the Area Under the Precision–Recall Curve (AUPRC). While AUROC measures the overall discriminative power, AUPRC is particularly informative in clinical datasets where class imbalances are prevalent. The general performance was also measured using accuracy.

(1) $$\mathrm{Accuracy}=\frac{TP+TN}{TP+TN+FP+FN}$$

where TP, TN, FP, and FN represent true positives, true negatives, false positives, and false negatives, respectively.

The length of stay (LOS) task is addressed as a regression problem to predict the remaining LOS after the initial 24-h observation. Patients who were discharged or died before 24 h were systematically excluded. The remaining LOS is $LOS_{total}-24$ h. The performance was quantified using the Mean Absolute Error (MAE), which provides a direct interpretation of the average error magnitude, and the Root Mean Squared Error (RMSE), which is more sensitive to large outliers:

(2) $$MAE=\frac{1}{n}\sum_{i=1}^{n}\left|y_{i}-{\hat{y}}_{i}\right|$$

(3) $$RMSE=\sqrt{\frac{1}{n}\sum_{i=1}^{n}{\left(y_{i}-{\hat{y}}_{i}\right)}^{2}}$$

where $y_{i}$ is the actual value, ${\hat{y}}_{i}$ is the predicted value, and *n* is the total number of observations.

A diverse set of algorithms was evaluated to capture both linear and nonlinear patterns. The hyperparameters for the tree-based and linear models were systematically optimized using an exhaustive grid search strategy combined with cross-validation across the training folds (GridSearchCV). Conversely, the Neural Network architectures were rigorously tuned with manually defined deepening strategies and dropout regularization to aggressively combat overfitting on the noisy empirical data. The final configurations of each algorithm used in the complete benchmark were as follows:

- Linear models: For classification tasks (mortality, septic shock), Logistic Regression was used with an L2 regularization penalty (Ridge) and a regularization constant (C) equal to 1.0 to mitigate overfitting. For the regression tasks, e.g., LOS, Lasso Regression was used, with L1 regularization (alpha = 1.0) to mitigate overfitting in the high-dimensional feature space.
- Random Forest: Decision Trees (n_estimators=100) were built, with max_depth = none (trees were fully expanded to the leaves) and a minimum sample split of 2 [28].
- XGBoost: Gradient boosting was configured with 200 estimators, a maximum tree depth of 6, and a learning rate of 0.1 [29].
- LightGBM: Optimized with 200 boosting iterations, a maximum depth of 6, and a learning rate of 0.1 [30].
- Long Short-Term Memory (LSTM): A Recurrent Neural Network (RNN) architecture was designed to capture long-term dependencies in time series data, used with 4 stacked layers, 128 hidden units per layer, and a dropout rate of 0.3 to heavily penalize over-parameterization. The model was trained using an Adam learning rate of 0.001 [31].
- Transformer: Neural Network (NN) with attention-based model with 4 encoder layers, 8 attention heads, a feed-forward dimension of 512, and a dropout rate of 0.3. The training was optimized using the Adam optimizer with a learning rate of 0.001.

A fixed random seed (seed = 42) was applied to all data splits to ensure consistent reproducibility across all models. Experiments were implemented in Python, utilizing the following tools: Pandas and NumPy for data manipulation; Scikit-learn for linear models, Random Forest, and evaluation metrics; PyTorch for deep learning architectures; the official XGBoost and LightGBM libraries for gradient boosting.

SHAP (Shapley Additive Explanations) analysis was employed to ensure the model’s transparency and clinical interpretability [32]. SHAP analysis provides three levels of explainability through three graphics: (1) overall importance of the feature (summary bar); (2) distribution of the feature’s impact among patients (bee swarm); (3) individual explanations of the predictions (waterfall plot). This multi-level transparency meets the key requirements of responsible AI use in healthcare applications.

## 3. Tasks and Results

Three types of tasks were prepared from the cohort: mortality, length of stay, and septic shock. This section describes the tasks, the test results and which features were most important to the results.

### 3.1. Mortality Task

The predictive efficacy was quantified using a combination of AUROC, AUPRC, and accuracy. Given the prevalence of class imbalance in the MIMIC-IV dataset, where septic events are significantly less frequent than non-events, the AUPRC serves as a critical metric for evaluating the trade-off between sensitivity and positive predictive value [33]. This was complemented by the AUROC to determine the global discriminative capacity and accuracy to assess the total proportion of correct classifications, ensuring a comprehensive validation of the model’s clinical utility. Table 5 presents the results of the tests for all algorithms, including the clinical scores SOFA and NEWS.

Comparative analysis of mortality prediction revealed that Gradient-Boosted Decision Trees (GBDTs) outperformed both traditional linear models and complex deep learning architectures. XGBoost achieved the highest performance across all metrics, with an AUROC of 0.871 and an AUPRC of 0.594. XGBoost demonstrated superior precision in identifying the positive class, as evidenced by its higher AUPRC.

The DeLong test confirmed that the results of the models (XGBoost, LightGBM, and Transformer) are statistically significant relative to the linear model. The gradient models demonstrated excellent calibration, with XGBoost achieving the lowest Brier Index (0.088) and LightGBM achieving the lowest ECE (0.022), indicating that their predicted probabilities closely matched the true empirical frequencies of patient mortality. This substantial performance superiority extends to the traditional clinical scores. The XGBoost model outperformed both the SOFA (AUROC: 0.730; accuracy: 0.810) and NEWS (AUROC: 0.697) baselines, confirming its incremental clinical value via the DeLong test (*p* < 0.001).

Deep learning models, such as Transformers and LSTM, achieve good results but are often outperformed by tree-based models on tabular data [34,35]. Unlike studies utilizing unstructured clinical text [36], our structured approach facilitates integration into standard workflows.

**Figure 2:**
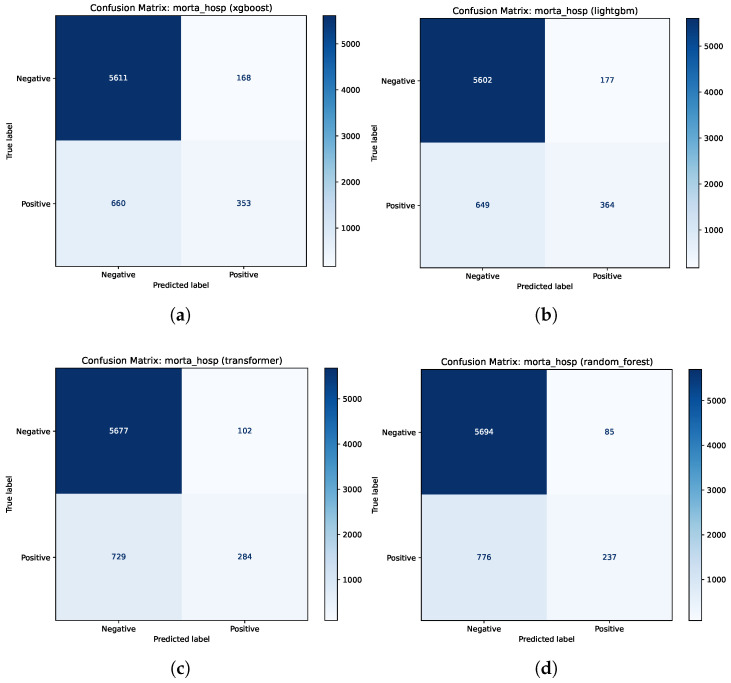
Confusion matrices for mortality task for the four best algorithms. Note: The layout follows the Scikit-Learn standard, where class 0 (negative/survivor) is presented in the first row/column and class 1 (positive/deceased) in the second. (**a**) Confusion matrix for XGBoost. (**b**) Confusion matrix for LightGBM. (**c**) Confusion matrix for Transformer. (**d**) Confusion matrix for Random Forest.

Figure 2 presents the confusion matrices for the four best algorithms in this task. The layout follows the Scikit-Learn Python library standard, where class 0 (negative/survivor) is presented in the first row/column and class 1 (positive/death) in the second. The confusion matrix can be interpreted as follows:

- Upper left quadrant: true negatives (TN), representing patients who survived (negative) and the model correctly predicted survival.
- Upper right quadrant: false positives (FP), representing patients who survived (negative) but the model incorrectly predicted death (positive). This situation is a false alarm that can be ignored.
- Lower left quadrant: false negatives (FN), representing patients who died (positive) but the model incorrectly predicted survival (negative). This is a critical error.
- Lower right quadrant: true positives (TP), representing patients who died (positive) and the model correctly predicted death.

The main diagonal (TN and TP) contains the correct predictions (starting with ’true’). The secondary diagonal (FN and FP) contains incorrect predictions (starting with ’false’). The first and second rows represent surviving and deceased patients, respectively.

The Random Forest model (Figure 2d) exhibited highly conservative behavior; while it minimized the lowest false positive count (85), it exhibited a clinically concerning rate of false negatives (776), failing to identify a significant proportion of patients at high mortality risk. The Transformer (Figure 2c) also leaned conservative, identifying only 284 true positives and maintaining a low false positive count (102) but missing 729 cases. Conversely, LightGBM (Figure 2b) identified the highest number of true positives (364), but at the cost of the highest rate of false positives (177), which could contribute to excess alarms in a clinical setting.

The XGBoost model (Figure 2a) exhibited the most balanced and consistent diagnostic performances. It successfully identified a high number of true positives (353) while maintaining better control over false positives (168) compared to LightGBM and achieved the highest overall discriminative ability, with AUROC (0.871) and AUPRC (0.594).

**Figure 3:**
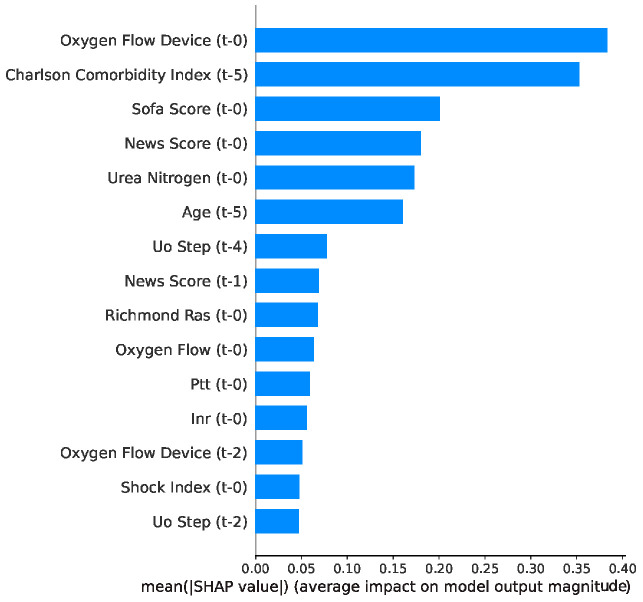
Top 15 features ranked by mean absolute SHAP value for mortality prediction for XGBoost.

Figure 3 shows a summary bar for XGBoost mortality prediction. The top 15 predictors are displayed along with their relevance to the model. According to the XGBoost algorithm, the Oxygen Flow Device (serving as a proxy for respiratory severity and need for intervention), Charlson Comorbidity Index, SOFA Score, and the NEWS Score were the main predictors of mortality.

On the predictor variables side, we have the measurement time, which was taken every 4 h, as explained earlier. For the mortality prediction task, the first 24 h were used to predict mortality. The interval T-0 represents the most recent measurements, taken 24 h after sepsis identification, while T-1 refers to measurements within 20 h of sepsis identification, and T-5 refers to measurements at the time of sepsis identification.

The SHAP bee swarm plot visualizes the global importance and directional effects of each feature. The variables were ranked vertically according to their overall influence on the models. The horizontal axis represents the SHAP value: positive deviations to the right of the vertical line indicate an increased probability, whereas negative values to the left of the vertical line suggest the opposite effect. The color gradients denote the original feature magnitudes: red for high values and blue for low values.

**Figure 4:**
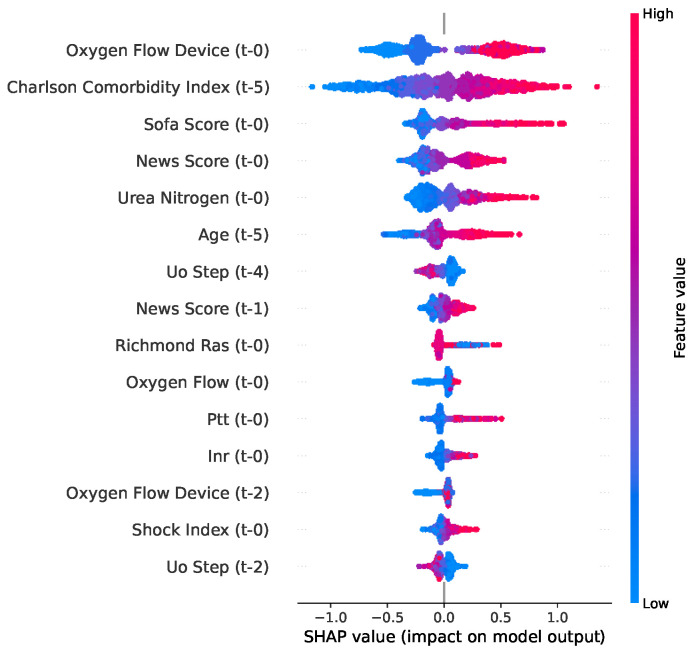
SHAP bee swarm plot for mortality used by the XGBoost model.

The SHAP analysis, presented in Figure 4, revealed that the Oxygen Flow Device, Charlson Comorbidity Index, and SOFA Score were the top predictors of mortality, where high values (red points) corresponded to positive SHAP values, indicating an increased risk. In contrast, Urine Output features (such as Uo Step) demonstrated an inverse relationship: high values, red color, shift towards the negative SHAP region, and reduced risk of mortality. Low values (blue), indicative of renal hypoperfusion and organ dysfunction, were strongly associated with an increased risk of mortality.

The SHAP waterfall plot shows the decision-making process of the model for a single instance, visualizing how each feature changes the prediction from the population baseline expected value to the final individual probability. Features are displayed in descending order of impact, where red bars indicate factors that increase the probability model and blue bars represent factors that decrease the probability.

**Figure 5:**
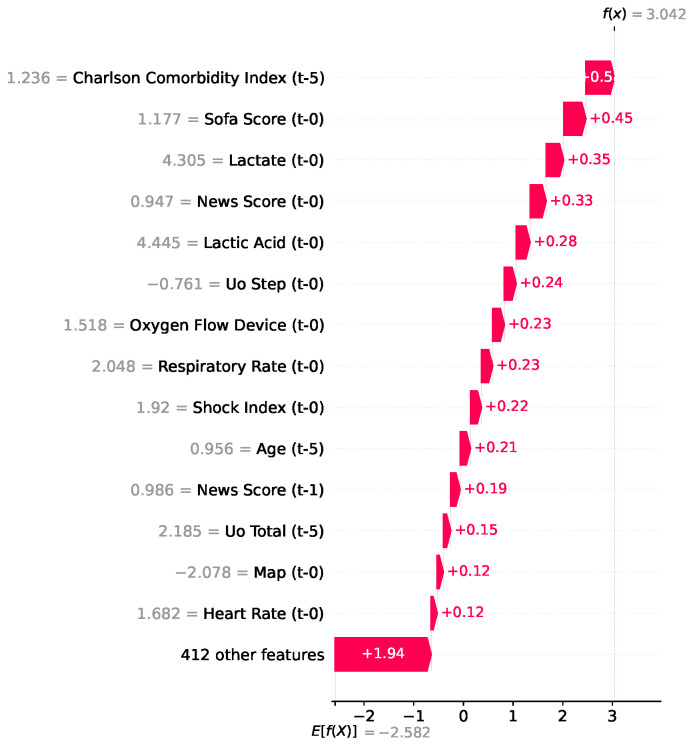
SHAP waterfall plot for mortality used by the XGBoost model.

In the case represented in Figure 5, the model estimated a remarkably high mortality risk. The final output value f(x)=3.042 (expressed in log-odds) was significantly higher than the population baseline of E[f(x)]=−2.582. This prediction was heavily driven upward by a combination of chronic conditions and acute deterioration of health. Specifically, the patient’s severe baseline comorbidities (Charlson Comorbidity Index, +0.50) and critical organ dysfunction markers (SOFA Score, +0.45; NEWS Score, +0.33) and elevated lactate level (+0.35) were the primary factors increasing the mortality estimate.

To demonstrate predictive fairness, a subgroup analysis of our top-performing model (XGBoost) for mortality task was conducted (Table 6). Both discrimination and precision-recall metrics remained remarkably stable between sexes (Male: AUROC 0.877, AUPRC 0.603; Female: AUROC 0.864, AUPRC 0.582), with closely overlapping 95% Confidence Intervals. Across age groups, dual-metric performance was robust in younger ranges (18–40: AUROC 0.935, AUPRC 0.646) and highly stable throughout core sepsis populations (41–65: AUROC 0.875; 66–80: AUROC 0.871), quantitatively confirming the absence of significant demographic bias.

### 3.2. Length of ICU Stay Task

For the regression task of predicting remaining length of stay (LOS), the results shown in Table 7 show the dominance of gradient-boosting algorithms over the others used in this test. LightGBM achieved the best performance, registering the lowest errors across all metrics (RMSE: 4.826 (±0.205); MAE: 2.541 (±0.035)), followed closely by XGBoost. The Transformer performed worse in this regression task, resulting in higher error rates (RMSE: 6.726 (6.309–7.131); MAE: 4.106 (3.976–4.228)). Therefore, complexity is not always associated with high accuracy.

Unlike the mortality task, traditional clinical scores such as SOFA and NEWS were not included as baseline values for this specific task, because this scores were designed for risk stratification and not for continuous temporal prediction, making them mathematically and clinically inadequate for direct regression with respect to remaining length of stay.

Clinically, the Mean Absolute Error (MAE) of approximately 2.6 days achieved by the LightGBM model represents a good parameter for resource planning, offering a reasonably accurate margin for bed management and discharge scheduling. There are no confusion matrices for LOS because this is a regression task (we are predicting a continuous number of days, e.g., 2.5 days) and not a classification (yes/no) task.

**Figure 6:**
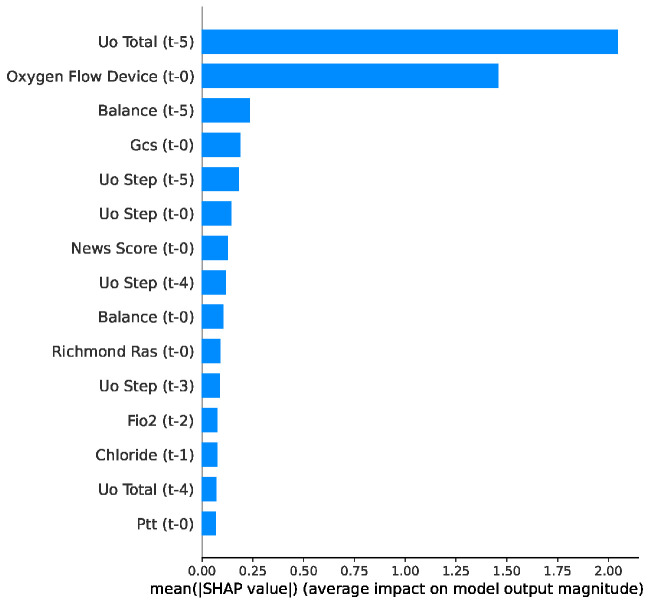
Top 15 features ranked by mean absolute SHAP value for LOS task used by LightGBM.

Feature importance analysis for the length of stay (LOS) regression task, Figure 6 reveals that indicators of organ dysfunction and therapeutic intensity are the primary drivers of hospitalization duration. length of stay (LOS) prediction is heavily influenced by dynamic physiological responses and resource utilization.

Renal function and fluid management were the primary determinants of LOS, with cumulative urine output (Uo Total) and fluid balance ranking first and third, respectively. Respiratory support also played a critical role, with the Oxygen Flow Device ranking second in importance. Furthermore, neurological status, as represented by the Glasgow Coma Scale (GCS) score, emerged as a significant predictor. These findings align with clinical reality, confirming that aggressive fluid resuscitation, respiratory severity, and neurological impairment are important drivers of prolonged hospitalization.

**Figure 7:**
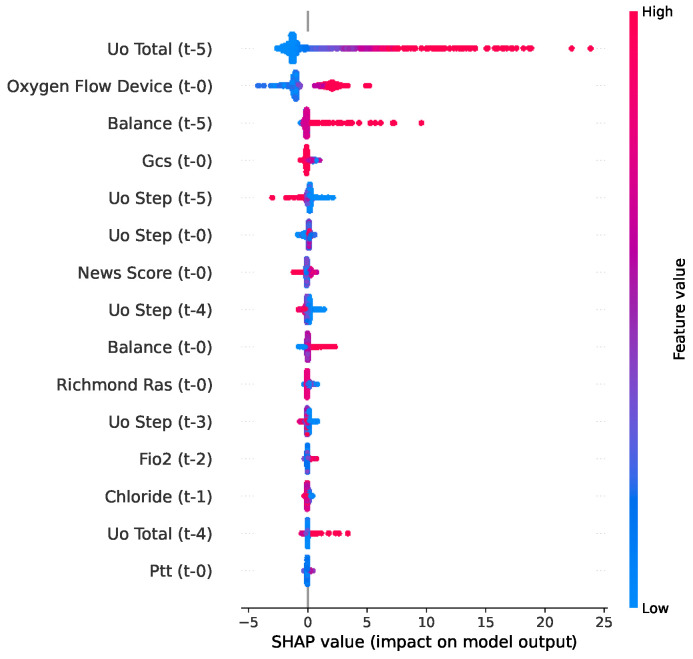
SHAP bee swarm plot for LOS used by the LightGBM model.

The SHAP bee swarm analysis for length of stay (LOS), presented in Figure 7, provides insight into how specific feature values influence hospitalization duration. The analysis revealed that the impacts of the top predictors, specifically Uo Total, Oxygen Flow Device, and fluid balance, were heavily skewed towards increasing LOS.

In the figure, the long tails of the red points extending to the right demonstrate that high values of these therapeutic and physiological intensity markers specifically drive predictions of significantly extended hospitalization, whereas lower values (blue) cluster near the baseline, indicating the standard recovery timelines. Furthermore, the high impact of an elevated fluid balance and sustained oxygen dependence accurately reflects the complex management of patients with prolonged organ dysfunction.

**Figure 8:**
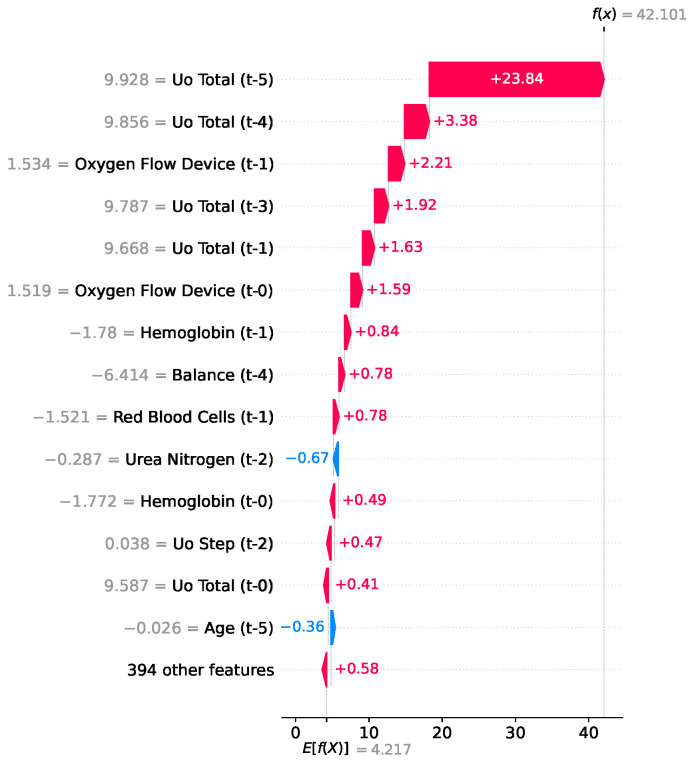
SHAP waterfall plot for LOS used by the LightGBM model.

In contrast to the expected LOS baseline of 5.2 days, the instance shown in Figure 8 presents an extreme outlier case that predicts a remarkably prolonged length of stay of 47 days. The dominant driver was cumulative urine output (Uo Total t-5), which alone added an unprecedented 23.84 days to the estimate, likely serving as a proxy for massive fluid resuscitation and complex physiological recovery following a severe shock. This primary factor is heavily reinforced by consecutive high measurements of urine output at subsequent time steps (t-4 and t-3) and the ongoing need for continuous respiratory support (Oxygen Flow Device at t-1 and t-0), painting a clinical picture of a patient requiring extensive critical care.

### 3.3. Septic Shock Task

For the septic shock prediction task (Table 8), the gradient-boosting algorithms demonstrated superior performance compared to the deep learning architectures. The gradient-boosting architectures XGBoost and LightGBM demonstrated superior and robust performance, with an AUROC of 0.950. The DeLong test confirmed that the performance gain of these tree-based models (XGBoost, LightGBM, and Random Forest) relative to the Logistic Regression baseline was statistically significant (p<0.001).

Furthermore, the gradient-boosting models exhibited excellent predictive calibration for this critical task. LightGBM achieved the lowest Expected Calibration Error (ECE of 0.011) and XGBoost achieved the lowest Brier Score (0.036), indicating that the probabilities generated for impending shock are reliable.

**Figure 9:**
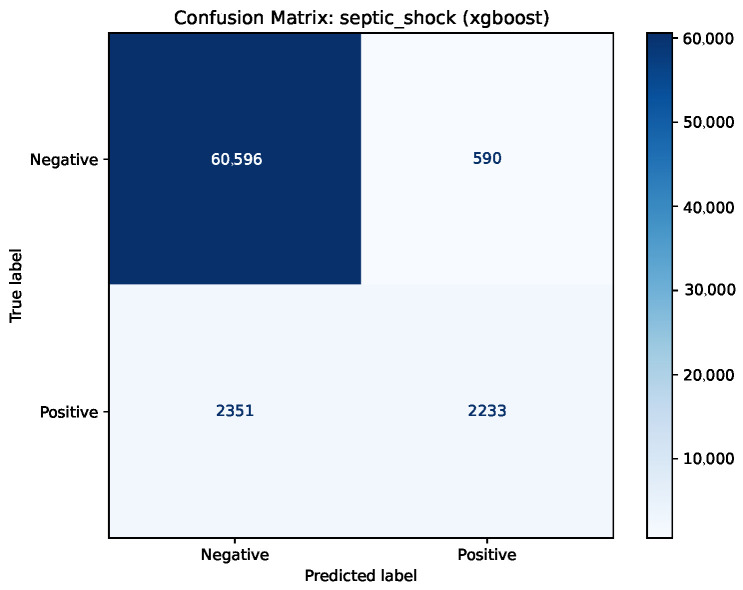
Confusion matrix for septic shock task using XGBoost.

According to the confusion matrix for the XGBoost model, presented in Figure 9, the model successfully excluded 60,596 instances (true negatives), generating only 590 false positives. This implies a high positive predictive value, indicating that, when the system signals a risk of septic shock, it is highly reliable.

However, the 2351 false negatives at the standard decision threshold (t=0.5) suggest that the model remained conservative. Given the robust AUPRC (0.753), this indicates that for clinical screening purposes, the decision threshold could be safely reduced to capture more high-risk patients without causing an uncontrollable increase in the number of false alarms.

Given the life-threatening nature of septic shock, the decision threshold could be lowered (e.g., to 0.2 or 0.3) to prioritize sensitivity. This adjustment would convert a significant portion of false negatives into true positives, ensuring earlier intervention for high-risk patients, although at the cost of a managed increase in alert frequency.

**Figure 10:**
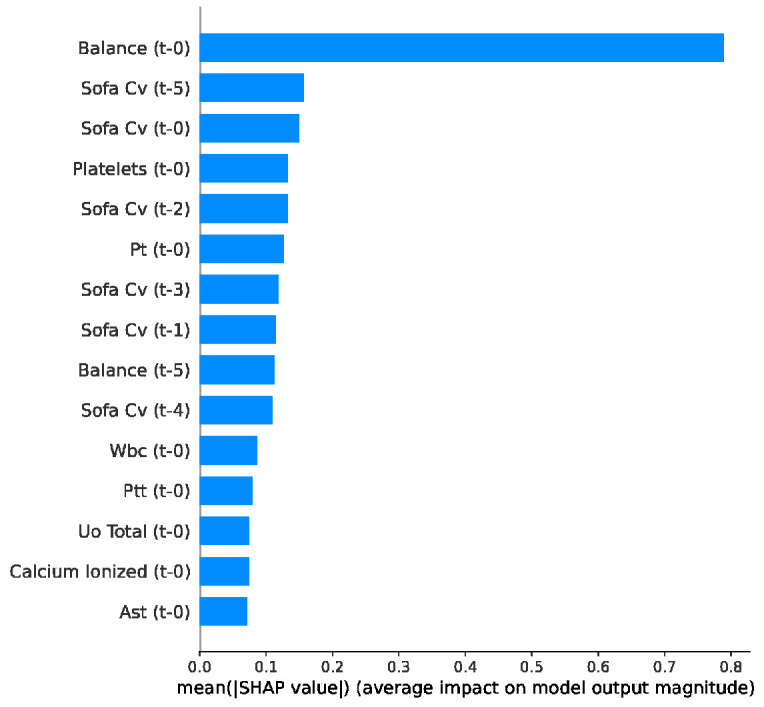
Top 15 features ranked by mean absolute SHAP value for septic shock prediction using XGBoost.

Global feature importance analysis (Figure 10) confirmed that the XGBoost model prioritizes complex physiological indicators for forecasting the onset of septic shock. Acute fluid accumulation, represented by fluid balance (Balance t-0), emerged as the most dominant predictor. This was closely followed by the cardiovascular component of the SOFA Score across multiple historical time steps (notably t-5 and t-0), validating the model’s focus on progressive hemodynamic instability. Furthermore, the high ranking of hematological markers (Platelets, PT, and WBC) reflects the systemic inflammatory and coagulation cascades that typically precede shock.

**Figure 11:**
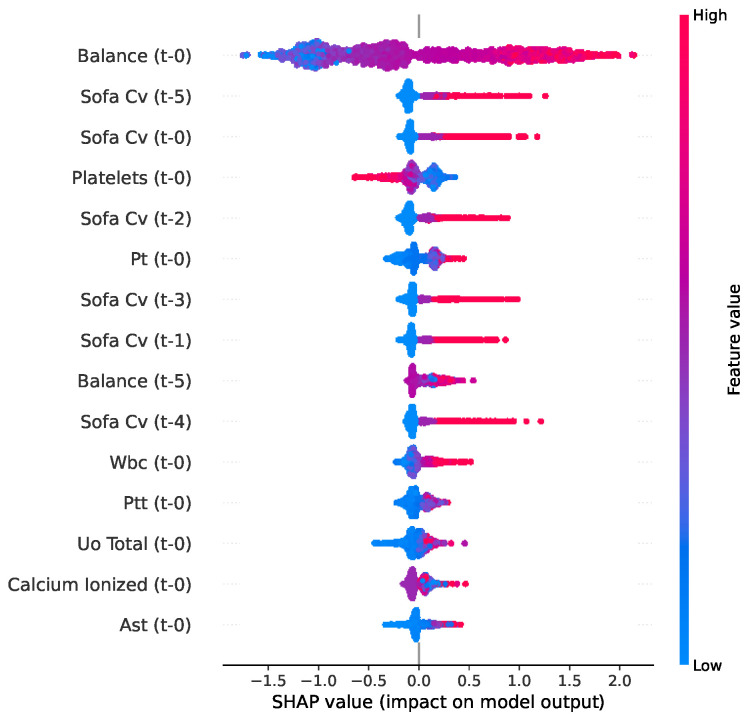
SHAP bee swarm plot for septic shock prediction used by the XGBoost model.

The SHAP bee swarm plot (Figure 11) extends this analysis by visualizing the directional impact of these clinical indicators. This reveals a distinct pattern for fluid balance (balance t-0) and the cardiovascular SOFA component (SOFA Cv), where elevated values (represented by red points) consistently shift the predictions toward a higher probability of septic shock.

**Figure 12:**
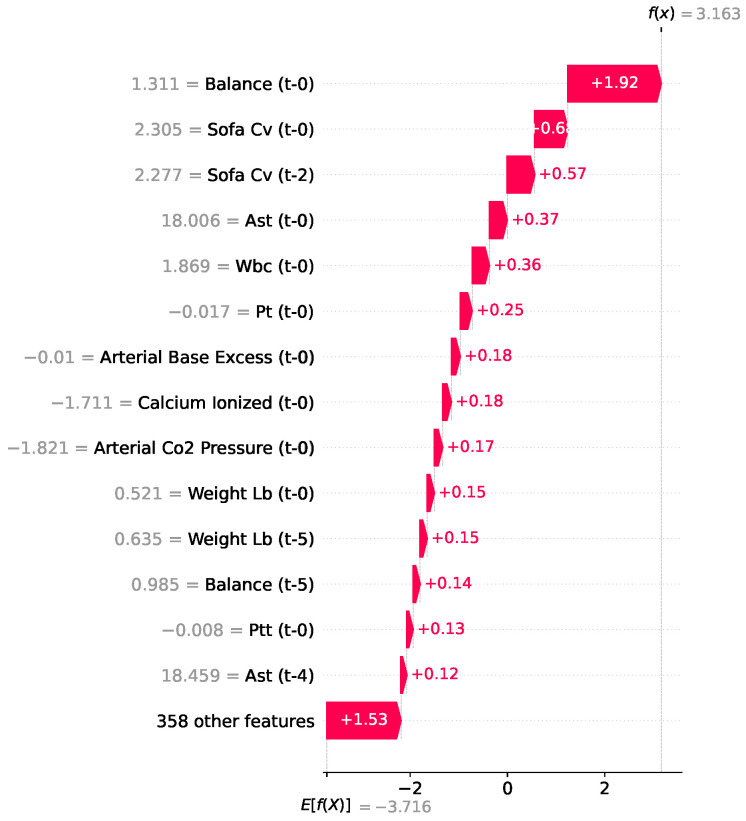
SHAP waterfall plot for septic shock prediction used by the XGBoost model.

In this case, as shown in Figure 12, the model evaluated a patient experiencing severe clinical deterioration. The baseline risk for the population was exceedingly low (E[f(X)]=−3.716), but the compounding acute physiological changes drove the final output to a remarkably high f(x)=3.163 (expressed in log-odds), indicating an imminent onset of septic shock.

This escalation in risk was driven by acute fluid retention (Balance t-0, +1.92) and progressive cardiovascular failure (SOFA Cv at t-0 and t-2, adding +0.60 and +0.57, respectively). These drivers were further compounded by markers of hepatic stress (AST) and systemic inflammation (WBC).

## 4. Discussion

For mortality prediction, we used a window of the first 24 h with data aggregated into 4-h timesteps to predict whether a patient would die from sepsis. The mortality prediction model achieved good performance with the XGBoost algorithm (AUROC = 0.871 and accuracy = 0.878), demonstrating that this algorithm works well in predicting the outcomes of patients with sepsis. SHAP analysis revealed that the Oxygen Flow Device, the Charlson Comorbidity Index, SOFA Score were the main predictors.

The regression task for predicting the length of ICU stay achieved good performance. An MAE = 2.541 for LightGBM is an acceptable error in the context of the number of days of hospitalization, representing a good parameter for resource planning, offering a reasonable margin for bed management and discharge scheduling. SHAP analysis showed that renal function, Oxygen Flow Device, and balance were determinants of LOS.

For septic shock prediction, we used the last 24 h to predict if the patient would experience septic shock in the next 24 h. The septic shock prediction model achieved excellent performance using the XGBoost algorithm (AUROC = 0.950 and accuracy = 0.955). SHAP analysis revealed that balance, SOFA Score, platelet count, prothrombin time, and white blood cell count were important predictors.

These results align with recent benchmarks demonstrating that tree-based models, such as XGBoost, consistently outperform deep learning architectures on tabular datasets [34,35]. While traditional linear and Logistic Regressions inherently provide immediate “glass-box” transparency via direct coefficient interpretation, an important advantage for front-line clinical trust, their rigid assumption of linear additivity inherently throttles their predictive ceiling. As demonstrated across the performance benchmarks, linear baselines struggle to map the highly irregular, nonlinear, and intensely interacting physiological trajectories characteristic of sepsis.

This mathematical limitation structurally justifies the necessary transition toward nonlinear gradient-boosting ensembles (GBDTs) and deep neural architectures. However, the adoption of these complex algorithms mandates their aggressive coupling with robust post hoc explainability frameworks, such as SHAP, to effectively recover the indispensable bedside transparency historically provided by simpler regression coefficients.

This study developed tasks related to sepsis prediction based on the responsible AI principles suggested in a recent editorial [19]. The MIMIC-IV database ensures that the data are aligned with ethical and safety principles. This is because it is free, has anonymized data, and has been approved by an ethics committee. Researchers are also required to complete an ethics course and make a formal request for data, declaring the purpose of its use. To access the data, the user agrees not to leak or provide the data to any third parties. The pipeline for extracting the sepsis cohort used, as well as the code for data processing, are also available.

The principles of explainability and transparency were satisfied. SHAP analysis allows physicians to better understand the model results, aiding their decision making. Regarding equity and bias mitigation, the statistical analyses of the cohort showed that the population was well-distributed in relation to sex and age group. Regarding mortality due to sepsis, the number of patients who died (5110) was much lower than the number of survivors (30,105) in this study.

To mitigate this, one of the chosen algorithms, XGBoost, handled the unbalanced data well. XGBoost uses the scale_pos_weight parameter to control the balance of weights between the positive and negative classes (binary classification). This makes it possible to increase the weight given to the minority (positive) class during training, penalizing the model more when it makes a mistake in that class.

The model used in this study contained 78 predictor variables, using a 24-h time window, with average extractions every 4 h. Considering the effect of a variable at a given moment, we have six time windows, resulting in 78 × 6 = 468 variables. The authors of [4] suggest using dynamic Bayesian networks (DBNs) instead of SHAP for explainability for physicians, which is very interesting. However, although DBNs are excellent for representing temporal dependencies and uncertainties, their complexity grows exponentially with an increase in the number of variables and time steps, resulting in a high computational cost.

The integration of SHAP values provides a necessary layer of trust in clinical applications [37]. This study demonstrates that the inherent opacity of complex algorithmic models can be effectively mitigated through structured explainability techniques, thereby facilitating their clinical implementation. A recent systematic review [38] highlighted that, while SHAP is the most dominant XAI method (used in 38% of studies), there is a significant gap in translating these interpretability tools into clinical practice because of their static and complex visualizations [39].

### Limitations

Although the MIMIC data are of high quality and have already been validated in academia, the database may contain some biases. The data models were trained from a single hospital center, the Beth Israel Deaconess Medical Center, limiting generalization to other hospitals and geographic regions, which may adopt different protocols for managing the disease.

Clinical protocols and demographic data vary across institutions. This study relied exclusively on the MIMIC-IV database. To attest to the robustness and clinical transferability of the algorithms, external validation on independent, multi-center cohorts is necessary. Future studies could test this model on other datasets, such as the eICU Collaborative Research Database [40]. Another limitation is that, while we used imputation, real-time clinical data often have gaps that can affect model inference.

The clinical utility of the model remains theoretical. We analyzed retrospective data and not real-time inputs. No prospective trials have been conducted to validate the tool in a hospital setting. Therefore, the actual impact on physician decision making and workflows remains unknown. Future work must assess how these predictions influence daily medical practice.

Another limitation concerns the analysis of antibiotic administration and microbiological cultures as indicators of suspected infection. Antibiotics may, in some situations, be administered empirically or erroneously, and positive cultures may reflect bacterial colonization rather than active infection. Although these factors introduce noise into the dataset, they also accurately reflect the reality and uncertainty of clinical decision making in the ICU.

Although SHAP provides state-of-the-art interpretability for intricate tree ensembles, it has inherent theoretical constraints. SHAP values are based on the mathematical assumption of feature independence, which is often not the case in clinical data due to natural physiological correlations.

When there is significant multicollinearity, SHAP may allocate importance across correlated variables, potentially reducing their individual rankings. Additionally, SHAP provides a post hoc linear approximation of the model’s logic for specific predictions, but it does not establish biological causality or accurately represent the complex, nonlinear decision boundaries of the models.

Finally, it is recognized that treating mortality and length of stay strictly as static classification and regression tasks represents a methodological limitation. Although the static 24-h early warning approach provides important prognostic information, it does not dynamically model time-to-event curves and does not consider competing clinical risks, such as discharge versus death. Future attempts at the integration of this framework should add formal survival analysis architectures, such as Cox Proportional Hazards or DeepSurv algorithms.

## 5. Conclusions

This study presents a comprehensive framework for predicting sepsis mortality, length of ICU stay, and septic shock using the MIMIC-IV v3.1 dataset. Developed under strict responsible AI principles, the study adhered to explainability, transparency, privacy, impartiality, fairness, and the TRIPOD-AI guidelines.

The test results showed that the algorithms performed well, and the SHAP plots identified features that were consistent with the task. The results demonstrated that tree-based models, specifically XGBoost, consistently outperformed deep learning architectures for tabular clinical data.

Beyond predictive accuracy, the high AUPRC addresses a critical challenge in intensive care: alarm fatigue. By minimizing false positives, the framework reduces the cognitive burden on medical staff, ensuring that alerts correspond to patients truly at risk of deterioration.

From an operational perspective, the accurate prediction of length of stay (MAE ≈ 2.5 days) offers tangible benefits for resource allocation, enabling better bed turnover planning and discharge scheduling, directly impacting the cost-effectiveness and operational efficiency of critical care units.

SHAP analysis allowed us to verify whether the model’s feature attributions aligned with established physiological expectations. By identifying clinical hallmarks such as respiratory support (Oxygen Flow Device), comorbidities (Charlson Index), and metabolic markers (Urea/Lactate) as top predictors, the model demonstrated alignment with established medical knowledge.

Future work will expand the model to predict specific interventions, such as the need for mechanical ventilation, and explore its integration with real-time web tools such as Shapash. This study demonstrates that high predictive power can coexist with interpretability. Although external validation is still needed, this framework provides a reliable method for the adoption of AI in intensive care units.

## Figures and Tables

**Table 1:** List of main predictor variables used in the models, grouped by clinical category.

Category	Features
Demographics	Age, gender, weight, height, readmission flag
Vital signs	Heart rate, respiratory rate, BP (systolic, diastolic, MAP), SpO_2_, temperature, shock index
Laboratory	Glucose, creatinine, BUN (urea nitrogen), potassium, sodium, chloride, magnesium, calcium (total, ionized, non-ionized), bicarbonate (HCO_3_), albumin, lactate
Exams	WBC count, hemoglobin, hematocrit, platelets, RBC count, coagulation (INR, PT, PTT)
Arterial blood gas	pH, PaO_2_, PaCO_2_, base excess, total CO_2_, PaO_2_/FiO_2_ ratio
Ventilation	Mechanical ventilation (binary), FiO_2_, PEEP, tidal volume, minute volume, peak insp. pressure, mean airway pressure, oxygen flow/device
Liver function	ALT, AST, bilirubin (total, direct)
Clinical scores	SOFA Score (total and components), NEWS Score, SIRS Score, Glasgow Coma Scale (GCS), RASS (sedation score), Charlson Comorbidity Index
Fluids, output	Fluid intake (total/step), urine (total/step), fluid balance, antibiotics (given, count, time since first)

**Table 2:** Cohort characteristics (extended from [20]).

Category	Value
Demographics	
Total Patients	35,215
Age (mean ± std)	65.4 ± 16.3
Sex (% female)	44.5%
Charlson Index (median [IQR])	5.0 [3.0–7.0]
Age Distribution (%)	
18–40	8.5%
41–65	38.1%
66–80	33.9%
>80	19.4%
BMI Distribution (%)	
Underweight (<18.5)	7.2%
Normal (18.5–24.9)	29.0%
Overweight (25–29.9)	26.5%
Obese (≥30)	37.3%
Clinical Outcomes	
Hospital mortality (%)	14.5%
90-day mortality (%)	25.1%
Length of stay (days)	5.1 ± 7.1
Readmission rate (%)	7.3%
Septic shock (%)	12.4%
Disease Severity (mean ± std)	
SOFA Score	5.5 ± 2.8
SIRS Score	1.5 ± 1.0
NEWS Score	6.06 ± 2.57
Glasgow Coma Scale	12.87 ± 3.76
Richmond Agitation–Sedation Scale	−0.94 ± 1.85
Interventions	
Mechanical ventilation (%)	35.1%
Vasopressor use (%)	16.9%
Antibiotics given (%)	66.3%
Patients receiving all 3 interventions (%)	23.3%
Fluid Management	
Mean fluid balance (mL)	−931.2 ± 8142.5
Fluid rate per 4h (mL)	458.9 ± 645.2
Cumulative fluid at 24 h (mL)	3017.3 ± 5566.5
Patients with negative fluid balance (%)	56.9%

**Table 3:** Statistical data for survivor and non-survivor groups.

Variable	Survivors	Non-Survivors	*p*-Value
	(n = 30,105)	(n = 5110)	
Demographics			
Age, median [IQR]	66.0 [55.0–77.0]	71.0 [60.0–81.0]	<0.001
Male Sex, n (%)	13,463 (44.7%)	2222 (43.5%)	0.103
Charlson Index, median [IQR]	5.0 [3.0–7.0]	6.0 [4.0–9.0]	<0.001
Clinical Scores (Admission)			
SOFA Score, median [IQR]	5.0 [3.0–7.0]	7.0 [5.0–9.0]	<0.001
NEWS Score, median [IQR]	6.0 [4.0–7.0]	7.0 [6.0–9.0]	<0.001
SIRS Score, median [IQR]	2.0 [1.0–2.0]	2.0 [1.0–3.0]	1.000
Vital Signs (Admission)			
Heart rate (bpm), median [IQR]	87.0 [75.7–100.8]	91.5 [78.7–106.2]	<0.001
MAP (mmHg), median [IQR]	79.2 [70.6–89.6]	75.6 [67.6–85.8]	<0.001
Respiratory rate (bpm), [IQR]	19.3 [16.3–23.0]	21.4 [18.0–25.2]	<0.001
SpO_2_ (%), median [IQR]	97.2 [95.2–99.0]	96.9 [94.7–98.8]	<0.001
Temperature (C), median [IQR]	36.8 [36.5–37.2]	36.8 [36.4–37.2]	1.000
Laboratory Values (Admission)			
Lactate (mmol/L), median [IQR]	1.6 [1.1–2.4]	2.0 [1.3–3.3]	<0.001
Creatinine (mg/dL), median [IQR]	1.0 [0.7–1.5]	1.3 [0.8–2.2]	<0.001
Bilirubin (mg/dL), median [IQR]	0.6 [0.4–1.4]	0.8 [0.4–2.2]	<0.001
Platelets (K/uL), median [IQR]	197.0 [137.0–271.0]	174.0 [103.0–259.0]	<0.001
WBC (K/uL), median [IQR]	10.4 [7.2–14.6]	12.4 [8.3–17.8]	<0.001
Outcomes			
LOS (h), median [IQR]	2.3 [1.3–5.0]	4.9 [2.5–9.7]	<0.001

**Table 4:** Task descriptions.

Task	Type	Approach	Metrics
Mortality	Binary Classification	Static (initial 24 h)	AUROC, AUPRC, accuracy
Remaining LOS	Regression	Static (initial 24 h)	RMSE, MAE
Septic Shock	Binary Classification	Dynamic (last 24 h)	AUROC, AUPRC, accuracy

**Table 5:** Results for mortality prediction. Note: * indicates statistical significance (p<0.001) compared to the linear model baseline using the DeLong test.

Model	AUROC (95% CI)	AUPRC (95% CI)	Accuracy (95% CI)	Brier Score	ECE
XGBoost	0.871 (0.861–0.882) *	0.594 (0.563–0.626)	0.878 (0.870–0.885)	0.088	0.029
LightGBM	0.869 (0.858–0.880) *	0.589 (0.558–0.621)	0.878 (0.870–0.886)	0.089	0.022
Transformer	0.866 (0.854–0.877) *	0.583 (0.552–0.614)	0.878 (0.870–0.885)	0.090	0.049
Random Forest	0.850 (0.838–0.862)	0.547 (0.515–0.580)	0.873 (0.865–0.881)	0.094	0.076
LSTM	0.847 (0.834–0.858)	0.529 (0.497–0.561)	0.868 (0.860–0.875)	0.104	0.135
Linear Model	0.843 (0.831–0.855)	0.530 (0.497–0.563)	0.870 (0.861–0.878)	0.095	0.040
SOFA Score	0.730 (0.718–0.742)	0.282 (0.246–0.320)	0.810 (0.793–0.827)	0.125	0.182
NEWS Score	0.697 (0.684–0.710)	0.252 (0.218–0.289)	0.801 (0.785–0.818)	0.134	0.210

**Table 6:** Subgroup fairness analysis (AUROC and AUPRC with 95% CI) of the XGBoost model for the mortality task.

Subgroup	Patients (n)	AUROC (95% CI)	AUPRC (95% CI)
Gender			
Female	15,671	0.864 (0.844–0.879)	0.582 (0.531–0.626)
Male	19,544	0.877 (0.863–0.890)	0.603 (0.562–0.643)
Age Cohort (Years)			
18–40	2993	0.935 (0.902–0.965)	0.646 (0.526–0.767)
41–65	13,417	0.875 (0.854–0.893)	0.579 (0.528–0.635)
66–80	11,938	0.871 (0.851–0.890)	0.619 (0.568–0.670)
>80	6832	0.826 (0.799–0.853)	0.586 (0.523–0.650)

**Table 7:** Results for remaining length of stay prediction (after 24-h observation.)

Model	RMSE (95% CI)	MAE (95% CI)	MSE
LightGBM	4.826 (±0.205)	2.541 (±0.035)	23.297
XGBoost	4.879 (±0.215)	2.584 (±0.038)	23.810
LSTM	4.918 (4.617–5.232)	2.618 (2.522–2.712)	24.186
Random Forest	4.934 (±0.172)	2.637 (±0.031)	24.349
Lasso Regression	5.465 (±0.284)	3.097 (±0.052)	29.876
Transformer	6.726 (6.309–7.131)	4.106 (3.976–4.228)	45.239

**Table 8:** Results for septic shock prediction. Note: * indicates statistical significance (p<0.001) compared to the Logistic Regression baseline based on the DeLong test.

Model	AUROC (95% CI)	AUPRC (95% CI)	Accuracy (95% CI)	Brier Score	ECE
XGBoost	0.950 (0.949–0.951) *	0.753 (0.748–0.758)	0.955 (0.954–0.956)	0.036	0.071
LightGBM	0.950 (0.949–0.951) *	0.738 (0.730–0.746)	0.953 (0.952–0.954)	0.037	0.011
Random Forest	0.938 (0.936–0.940) *	0.692 (0.686–0.698)	0.946 (0.945–0.947)	0.041	0.098
Logistic Regression	0.896 (0.894–0.898)	0.485 (0.478–0.492)	0.932 (0.931–0.933)	0.053	0.058
LSTM	0.878 (0.875–0.881)	0.516 (0.507–0.524)	0.930 (0.929–0.931)	0.060	0.188
Transformer	0.667 (0.663–0.670)	0.187 (0.182–0.192)	0.921 (0.920–0.922)	0.069	0.040

## Data Availability

The data that support the findings of this study are derived from the MIMIC-IV database, which is publicly available at https://physionet.org/content/mimiciv/3.1/ (accessed on 20 October 2025) for credentialed number researchers who complete the required data use agreement. The source code is available at: https://github.com/thiagoqo/resp-mimic-sepsis (accessed on 15 January 2026).

## Abbreviations

The following abbreviations are used in this manuscript:

AI	Artificial intelligence
AUC	Area Under the Curve
AUPRC	Area Under the Precision–Recall Curve
AUROC	Area Under the Receiver Operating Characteristic curve
BMI	Body Mass Index
BUN	Blood Urea Nitrogen
CCI	Charlson Comorbidity Index
CI	Confidence Interval
CRP	C-Reactive Protein
DL	Deep Learning
ECE	Expected Calibration Error
EHR	Electronic Health Record
DNN	Deep Neural Networks
FiO_2_	Fraction of Inspired Oxygen
FN	False negative
FP	False positive
GBDT	Gradient-Boosted Decision Trees
GCS	Glasgow Coma Scale
HR	Heart Rate
LightGBM	Light Gradient-Boosting Machine
LIME	Local Interpretable Model-agnostic Explanations
LOS	length of ICU stay
LR	Logistic Regression
LSTM	Long Short-Term Memory
MAE	Mean Absolute Error
MAP	Mean Arterial Pressure
MIMIC	Medical Information Mart for Intensive Care
ML	Machine learning
MLMs	Machine learning models
NEWS	National Early-Warning Score
NN	Neural Network
O_2_	Oxygen Flow Rate
PaCO_2_	Partial Pressure of Arterial Carbon Dioxide
PaCO_2_/FiO_2_	Partial Pressure of Arterial Oxygen/Fraction of Inspired Oxygen
pH	Arterial pH
ICU	Intensive care unit
qSOFA	Quick Sequential Organ Failure Assessment
RASS	Richmond Agitation–Sedation Scale
RMSE	Root Mean Squared Error
RNN	Recurrent Neural Network
RR	Respiratory Rate
SaO_2_	Arterial Oxygen Saturation
SBP	Systolic Blood Pressure
SHAP	Shapley Additive Explanations
SIRS	Systemic Inflammatory Response Syndrome
SOFA	Sequential Organ Failure Score
TN	true negative
TP	true positive
SpO_2_	Peripheral Oxygen Saturation
WBC	White Blood Cell count
WHO	World Health Organization
xAI	Explainable artificial intelligence
XGBoost	eXtreme Gradient Boosting

## Appendix A. Predictor Variables

Table A1 lists the 78 predictor variables used in the experiments, in alphabetical order.

**Table A1:** List of 78 predictor variables in alphabetical order.

abx_given (Antibiotics Given—Binary)	minute_volume (Minute Volume)
age (Age)	news_score (National Early-Warning Score)
albumin (Albumin)	num_abx (Number of Antibiotics)
alt (Alanine Aminotransferase)	oxygen_flow (Oxygen Flow Rate)
arterial_base_excess (Arterial Base Excess)	oxygen_flow_device (Oxygen Flow Device)
arterial_co2_pressure (PaCO_2_)	peak_inspiratory_pressure (Peak Inspiratory Pressure)
arterial_o2_pressure (PaO_2_)	peep (Positive End-Expiratory Pressure)
ast (Aspartate Aminotransferase)	pf_ratio (PaO_2_/FiO_2_ Ratio)
balance (fluid balance)	ph_arterial (pH—Arterial)
bilirubin_direct (Direct Bilirubin)	platelets (Platelets Count)
bilirubin_total (Total Bilirubin)	potassium (Potassium)
calcium_ionized (Ionized Calcium)	pt (Prothrombin Time)
calcium_non_ionized (Non-ionized Calcium)	ptt (Partial Thromboplastin Time)
calcium_total (Total Calcium)	re_admission (Readmission Flag)
charlson_comorbidity_index (Charlson Index)	red_blood_cells (RBC Count)
charttime (Chart Time)	respiratory_rate (Respiratory Rate)
chloride (Chloride)	richmond_ras (RASS Scale)
creatinine (Creatinine)	sbp_arterial (Systolic BP—Arterial)
dbp_arterial (Diastolic BP—Arterial)	shock_index (Shock Index)
fio2 (Fraction of Inspired Oxygen)	sirs_score (SIRS Score)
fluid_step (Fluid Intake per Step)	sodium (Sodium)
fluid_total (Total Fluid Intake)	sofa_cns (SOFA CNS Component)
gcs (Glasgow Coma Scale)	sofa_coag (SOFA Coagulation Component)
gender (Gender)	sofa_cv (SOFA Cardiovascular Component)
glucose (Glucose)	sofa_liver (SOFA Liver Component)
hco3 (Bicarbonate)	sofa_renal (SOFA Renal Component)
heart_rate (Heart Rate)	sofa_resp (SOFA Respiratory Component)
height_cm (Height in cm)	sofa_score (Total SOFA Score)
height_inch (Height in inches)	spo2 (Pulse Oximetry Saturation)
hematocrit (Hematocrit)	temp_C (Temperature in Celsius)
hemoglobin (Hemoglobin)	temp_F (Temperature in Fahrenheit)
hours_since_first_abx (Hours since First Abx)	tidal_volume (Tidal Volume)
inr (International Normalized Ratio)	total_co2 (Total CO_2_)
lactate (Lactate)	uo_step (Urine Output per Step)
lactic_acid (Lactic Acid)	uo_total (Total Urine Output)
magnesium (Magnesium)	urea_nitrogen (BUN)
map (Mean Arterial Pressure)	wbc (White Blood Cells Count)
mean_airway_pressure (Mean Airway Pressure)	weight_kg (Weight in kg)
mechvent (Mechanical Ventilation—Binary)	weight_lb (Weight in lb)
